# Random Item Response Data Generation Using a Limited-Information Approach: Applications to Assessing Model Complexity

**DOI:** 10.1017/psy.2025.10017

**Published:** 2025-05-21

**Authors:** Yon Soo Suh, Wes Bonifay, Li Cai

**Affiliations:** 1 NWEA within HMH; 2 University of Missouri, Columbia, MO, USA; 3 University of California, Los Angeles, Los Angeles, CA, USA

**Keywords:** fitting propensity, item response theory, limited-information methods, model complexity, sequential importance sampling

## Abstract

Fitting propensity (FP) analysis quantifies model complexity but has been impeded in item response theory (IRT) due to the computational infeasibility of uniformly and randomly sampling multinomial item response patterns under a full-information approach. We adopt a limited-information (LI) approach, wherein we generate data only up to the lower-order margins of the complete item response patterns. We present an algorithm that builds upon classical work on sampling contingency tables with fixed margins by implementing a Sequential Importance Sampling algorithm to Quickly and Uniformly Obtain Contingency tables (SISQUOC). Theoretical justification and comprehensive validation demonstrate the effectiveness of the SISQUOC algorithm for IRT and offer insights into sampling from the complete data space defined by the lower-order margins. We highlight the efficiency and simplicity of the LI approach for generating large and uniformly random datasets of dichotomous and polytomous items. We further present an iterative proportional fitting procedure to reconstruct joint multinomial probabilities after LI-based data generation, facilitating FP evaluation using traditional estimation strategies. We illustrate the proposed approach by examining the FP of the graded response model and generalized partial credit model, with results suggesting that their functional forms express similar degrees of configural complexity.

## 1 Introduction

Statistical model evaluation requires balancing goodness-of-fit (GoF) to observed data and generalizability to future/unseen data. Achieving this balance is not always straightforward, as GoF and generalizability are both affected by model complexity, or the capacity of the model to fit diverse data patterns (Pitt & Myung, 2002). In applications of statistical modeling and inference, there is an over-reliance on GoF to the observed data (especially in the social sciences; Roberts & Pashler, 2000), and, consequently, the problem of complexity is often downplayed or ignored. When complexity is considered, it is routinely quantified using relative fit statistics like Akaike Information Criterion (AIC) and Bayesian Information Criterion (BIC), which penalize the GoF when it comes at the cost of many model parameters; but this *parametric complexity* is just one of multiple factors that influence the overall model complexity. Models may also exhibit *configural complexity*, which is driven by the arrangement of the parameters in the model’s functional form. Taken together, two or more models with the same parametric complexity may differ in configural complexity, such that one model is inherently more likely to fit any given data pattern (Bonifay & Cai, 2017; Falk & Muthukrishna, 2023; Myung et al., 2005; Preacher, 2006; Romeijn, 2017).

**Figure 1:**
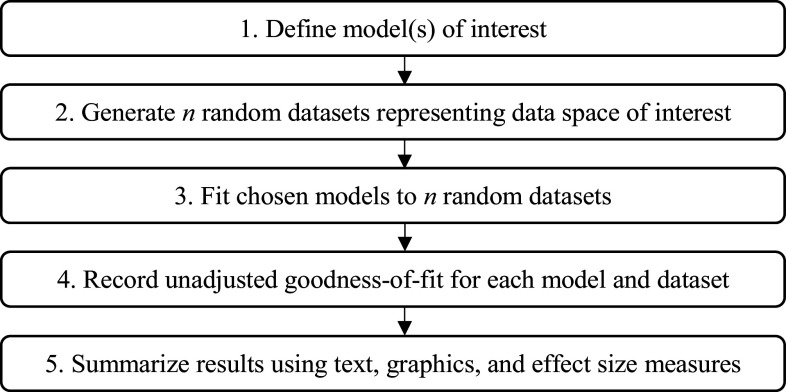
Procedure for assessing fitting propensity.

Unfortunately, the detection of configural complexity requires more than just tallying parameters. Preacher (2006) introduced *fitting propensity* (FP) analysis as a method by which to uncover the configural complexity of a statistical model. In general, FP analysis follows the procedure outlined in Figure 1 (Falk & Muthukrishna, 2023). First, the researcher defines the model(s) of interest. Then, a large number of datasets are randomly and uniformly sampled from the complete space of all possible data. The candidate model(s) are then fit to all datasets, and the unadjusted GoF of each model to each dataset is recorded. A summary of this process, in textual, graphical, and statistical output, describes the propensity of each model to fit well to any given data pattern. If the model fits a large proportion of the generated patterns, it is said to have high FP; in such a case, good fit is unsurprising, so evaluation of such a model should place minimal weight on the GoF statistics. Conversely, if the model fits only a small proportion of the data space, then good fit is a surprising outcome, so model evaluations can place more weight on the GoF statistics. FP analysis is especially insightful when multiple models with strong GoF statistics are under evaluation, as one can select the model that is inherently less likely to fit well (and thus more likely to represent the generalizable regularity in the data; Vitányi & Li, 2000).

Preacher (2006) explored FP analysis in structural equation modeling (SEM) by evaluating the performance of several sets of models in the complete space of all possible continuous data. He demonstrated, for example, that when a factor model and an autocorrelation model (each with 11 free parameters) were fit to 10,000 random correlation matrices, the factor model exhibited good fit far more often. By controlling for the number of parameters, Preacher illustrated that functional form can imbue a model with configural complexity so that its GoF becomes more of a statistical artifact than an informative model evaluation metric.

Bonifay and Cai (2017) extended FP analysis to the categorical data space by examining a set of item response theory (IRT) models, as detailed below. In IRT, the complete data space consists of all possible response patterns for a set of items. Generation of this data space is achievable for a limited number of items under the conventional full-information (FI) approach of the multinomial framework (as in Bonifay & Cai, 2017), but it typically involves a high-dimensional discrete space that renders uniform random sampling and model fitting of all response patterns computationally infeasible. Consequently, further study of the IRT model FP has been constrained by the number and types of items.

To address these limitations, we propose a limited-information (LI) approach, as suggested by numerous scholars over the decades, including Bolt (2005) and earlier references therein. LI methods typically use information only up to item pairs (i.e., first- and second-order margins; e.g., Bartholomew & Leung, 2002; Reiser, 1996), which can be obtained by collapsing the full item response patterns into contingency tables of consecutive lower-order margins (e.g., Cai et al., 2006; Maydeu-Olivares & Joe, 2005, among others). Following this logic, we propose an efficient data generation algorithm that simulates only the univariate and bivariate margins for a set of items, thereby satisfying the second step of FP analysis. Our method is founded on classical literature about sampling contingency tables with fixed margins, combined with the sequential importance sampling (SIS) algorithm. Through these techniques, the dimensionality of the complete data space is then brought down to the bivariate margins, which significantly reduces the number of response probabilities that need to be generated. To fulfill the third step of FP analysis, we show how the iterative proportional fitting procedure (IPFP) allows one to use standard FI maximum likelihood methods to fit IRT models by reconstructing multinomial probabilities from the univariate and bivariate margins. Overall, the computational gain from these LI strategies paves the way for simulating more advanced IRT modeling schemes that are disallowed under the FI approach due to unmanageable numbers of item response patterns.

This article is organized as follows. We begin by providing an overview of FP and the evaluation of IRT models using FP. We then discuss the complete data space for IRT models and the corresponding number of item response probabilities to be randomly and uniformly sampled under both FI and LI methods. Next, we detail the geometry of the complete categorical data space, which forms the theoretical basis for our novel item response generation algorithm. We then present our proposed algorithm, demonstrating its effectiveness, computational efficiency, and suitability, while shedding light on the process of sampling from the data space defined by lower-order margins. Lastly, we illustrate the application of our algorithm, along with IPFP-based estimation (Deming & Stephan, 1940), to the investigation of the FP of two IRT models for polytomous data.

## 2 Fitting propensity

### 2.1 Fitting propensity

Box (1979) stated that “all models are wrong, but some are useful.” Three quantifiable measures of a model’s usefulness include GoF, generalizability, and complexity (Myung et al., 2005). GoF represents a model’s ability to fit a particular dataset, and generalizability is a measure of a model’s predictive accuracy regarding future and/or unseen replication samples. Both are impacted by model complexity, as defined earlier. Model evaluation is, therefore, an act of balancing GoF and generalizability so that one selects a model capturing maximal regularity and minimal noise in the data.

One path toward achieving this balance is to frame complexity as FP, which is grounded in the information-theoretic principle of minimum description length (MDL; Rissanen, 1978). According to MDL, the best model is that which compresses the complete data space using a concise algorithmic description, or code. The MDL principle is the basis for several model complexity criteria, including stochastic information complexity (Rissanen, 1989), Fisher information approximation (Rissanen, 1996), Information Complexity Criterion (ICOMP) (Bozdogan, 1990), and others.

For the present study, the most relevant formulation of MDL is given by Rissanen’s (2001) normalized maximum likelihood (NML):
(1)

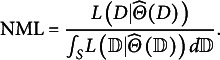



Here, 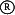 is the observed data, 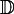 is all possible data from space 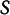 , and 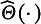 contains the maximum likelihood parameter values for a given dataset. Thus, NML compares the model’s fit to the observed data relative to its fit to any possible data. Unfortunately, integration across the complete data space is practically intractable for many model classes, including SEM and IRT (Preacher, 2006; Bonifay & Cai, 2017), thus necessitating the role of simulation-based MDL approximation via FP analysis. Like NML, FP is based on the premise that some models simply have the potential to fit a wide range of data patterns. In that light, FP can be described as the inverse of parsimony: higher FP indicates that a model is less parsimonious.

**Figure 2:**
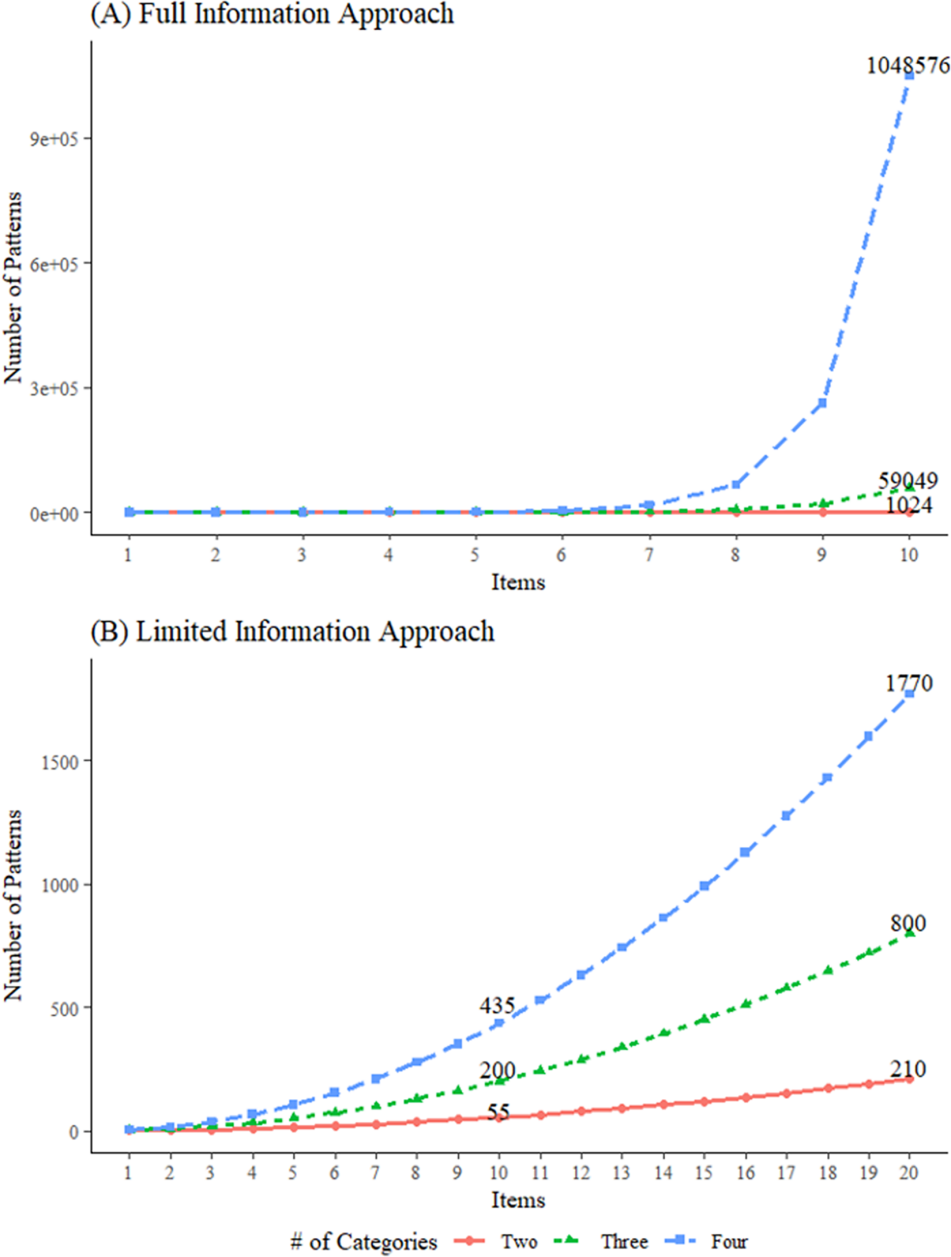
Number of data patterns to generate under the full-information versus limited-information approaches.

### 2.2 Fitting propensity and item response theory

Although one can examine FP for a single model, it is especially beneficial for comparing multiple models in terms of how well each fits any given pattern from the space of all possible data. Following the logic of Preacher (2006), Bonifay and Cai (2017) used the procedure outlined in Figure 1 to examine whether five widely applied dichotomous IRT models differed in configural complexity: an exploratory item factor analytic model, a (confirmatory) bifactor model, two diagnostic classification models, and a unidimensional 3-parameter logistic (3PL) model. Their first four models were specified to have the same parametric complexity (20 parameters each), but different functional forms. The unidimensional 3PL model had greater parametric complexity (21 parameters), but a seemingly less complex functional form.

Working within the conventional FI framework, they defined the complete data space using all cell probabilities of the multinomial model, where each cell corresponded to one item response pattern. In the context of IRT, randomly and uniformly sampling from this data space translates to generating probability vectors for every possible response pattern, ensuring they are uniformly distributed and sum to 1.0. Bonifay and Cai (2017) generated 1,000 sets of such response patterns based on the simplex sampling method first proposed by Rubin (1981), fit all five models to each dataset, and summarized the results using Bartholomew and Leung’s (2002) Y2/*N* unadjusted fit index. They found that the exploratory factor model and the bifactor model both had, by far, the highest FPs, whereas the unidimensional 3PL model exhibited the lowest FP (despite its extra parameter). Their results underscored the importance of considering functional form, providing further evidence that model complexity in IRT, as in SEM, cannot be fully understood simply by counting free parameters.

However, the main limitation of their study was that the number of all possible response patterns grows exponentially with the number of items. In traditional FI-based methods under the multinomial framework, the total number of response patterns is equal to 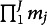 , where 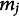 refers to the number of categories for an item 
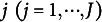
 . As shown in Figure 2A, Bonifay and Cai’s (2017) sampling method becomes computationally infeasible as the number of items and response categories increase, which limits the range of models for which FP can be evaluated.

To address this problem, we propose a LI-based approach that can accommodate a wide variety of IRT models and/or a large number of items. Our approach is based on two premises: that item response probabilities can be organized into contingency tables and that IRT models can be defined on the marginal moments of the multivariate Bernoulli (MVB) distribution. Instead of simulating datasets as full multinomial contingency tables where each cell denotes the frequency of a specific response pattern, we simulate data for only the lower-order margins. Accordingly, only 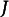 first-order margins and 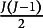 second-order margins are needed, where *J* denotes the number of items. Thus, the total number of probabilities is 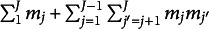 , which provides a significant reduction relative to sampling the full multinomial probabilities. This is clearly shown in Figure 2B, where the number of lower-order margins that must be simulated follows a lower-order polynomial in the number of items, in contrast to the exponential increase in Figure 2A.

## 3 Contingency tables and item response theory

### 3.1 Two representations of item response theory models

Contingency tables of item response data have two equivalent representations: (1) the *cells representation* based on cell probabilities of the item-by-item cross-classifications, and (2) the *margins representation* based on the marginal moments (Maydeu-Olivares & Joe, 2014). The former follows the familiar multinomial distribution theory, while the latter follows the MVB framework (Bahadur, 1961; Teugels, 1990). Both approaches generalize to tables of any size or categories. Suppose that we have *J* items and *N* individuals (indexed 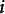 ). Let 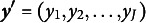 be the vector of *J* variables 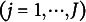 , where each variable has 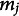 response alternatives. Responses to the items are realized as a *J*-way contingency table with a total of 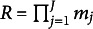 cells corresponding to the possible response vectors 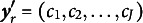 , where 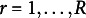 and 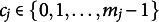 .

Let us consider only dichotomous item responses, where 0 = incorrect and 1 = correct. In the cells representation, 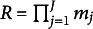 is equal to 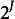 , with each cell representing one of the 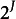 item response patterns, 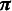 . Each of these item response patterns 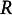 can be considered as a random *J*-vector 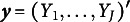 of (typically codependent) Bernoulli random variables for which 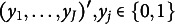 is a realization. The joint distribution of the MVB random vector 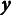 is then
(2)

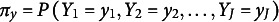



In the margins representation, the 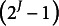 -vector 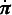 of joint moments of the MVB distribution has the partitioned form 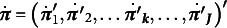 , where the dimension of vector 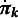 is 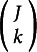 . 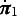 indicates the set of all *J* univariate or first-order marginal moments, where 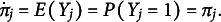 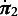 denotes the set of 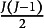 bivariate or second-order marginal moments, 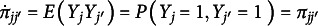 for all distinct 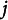 and 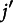 satisfying 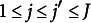 . The joint moments are defined in this manner up to the last one, 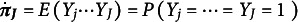 , with a dimension of 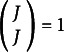 (Cai et al., 2006).

Consider a 2 × 2 table for a pair of dichotomous items, which represents the smallest multivariate categorical data example, as shown in Table A1 in the Appendix. The cells representation consists of four cell probabilities that sum to one. The margins representation uses three moments: two means, 
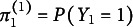
 and 
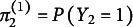
 and the cross product 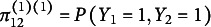 . There is a one-to-one relationship between the representations that is invertible irrespective of the number of categorical variables (Teugels, 1990).

In sum, generating item responses from the lower-order moments (i.e., item pairs) is equivalent to randomly sampling from two-way contingency tables with margin constraints. In this article, we adopt the latter strategy by introducing a random categorical data generation algorithm based on the MVB framework and the lower-order margins. Before we present our algorithm, however, we consider the geometric interpretations of contingency tables, specifically those with fixed margins, which hold the key to understanding how to randomly sample from the complete space of such tables (Diaconis & Efron, 1985; Fienberg, 1970; Fienberg & Gilbert, 1970; Nguyen & Sampson, 1985; Slavković & Fienberg, 2009).

### 3.2 Geometry of 2 × 2 contingency tables with fixed margins

**Figure 3:**
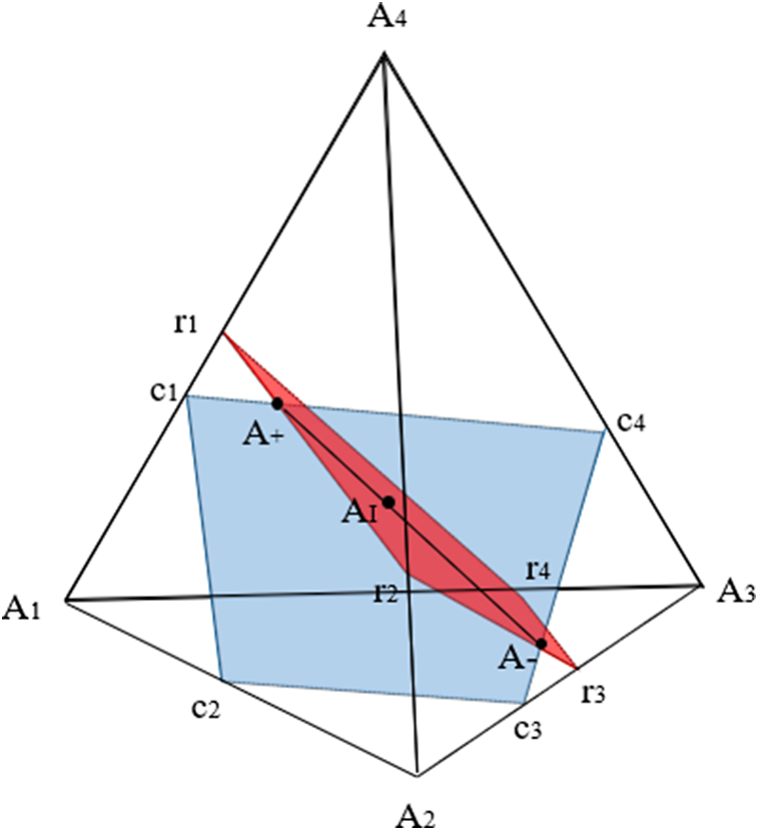
Tetrahedron depicting a 2 × 2 contingency table with fixed margins. *Note:* Adapted from Nguyen and Sampson (1985).

For explanation purposes and ease of graphical representation, we consider two univariate binary variables 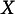 and 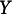 that can refer to any item pair 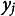 and 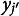 . The joint probability mass function (PMF) for any item pair is a 2 × 2 table of cell probabilities 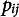 , where 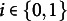 and 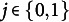 are drawn from a bivariate Bernoulli distribution. The set 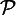 of all 2 × 2 PMF matrices 
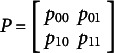
 can be geometrically represented within a three-dimensional probability simplex, which we denote as 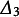 . As shown in Figure 3, when using barycentric coordinates, 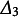 takes the form of a regular tetrahedron with vertices 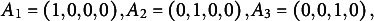 and 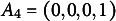 (Slavković & Fienberg, 2009). A tetrahedron has four faces, or two-dimensional simplices, each of which can be defined by combinations of three of the four vertices: Face 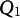 is defined by 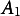 , 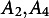 ; 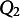 by 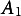 , 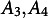 ; 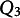 by 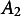 , 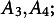 and 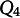 by 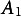 , 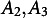 . There is a one-to-one correspondence between points 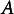 of the simplex, with coordinates 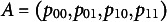 , and the 2 × 2 PMF matrices. The points 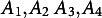 refer to the four extreme PMF matrices in which one cell has 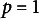 and all other cells have 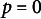 .

Let 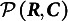 be the set of all 2 × 2 PMF matrices with fixed row marginal probability vector 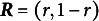 and column marginal probability vector 
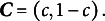
 By fixing one of the cell probabilities, such as 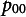 , a PMF matrix *P* of 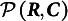 is completely determined as
(3)

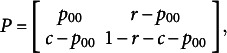

 which reflects point 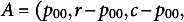 
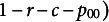
 in the simplex 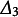 . Let two planes 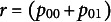 and 
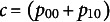
 intersect 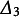 so that 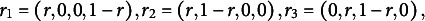 and 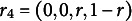 ; and 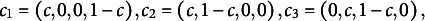 and 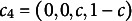 . Geometrically, each plane describes the set of points defined by a single fixed marginal (i.e., the red and blue planes in Figure 3).

As shown in the figure, the set 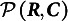 is then the line segment given at the intersection of these planes, which determine the set of PMF matrices that satisfy the marginal constraints set by both 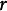 and 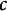 . The two extreme points of the line segment are called the upper Fréchet bound 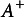 and lower Fréchet bound 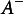 , where
(4)

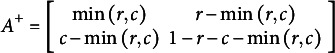

 and
(5)

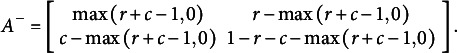



The independence model for a 2 × 2 table is also a matrix of 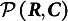 denoted by
(6)

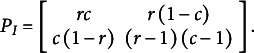



**Figure 4:**
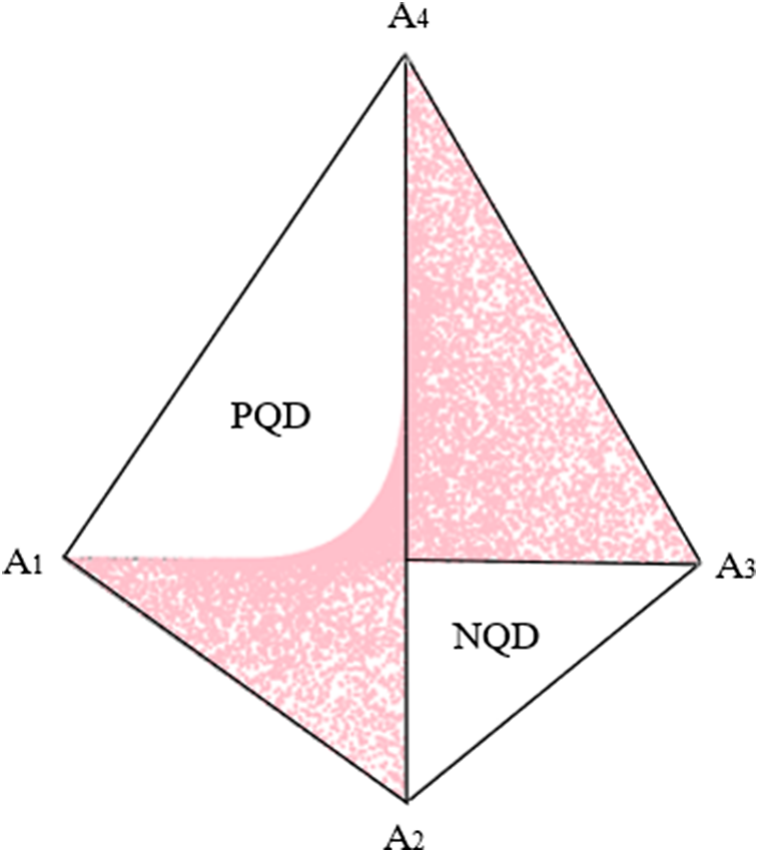
Surface of independence. *Note:* Adapted from Nguyen and Sampson (1985).

This is equivalent to the point 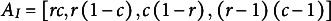 depicted in Figure 4 (Fienberg & Gilbert, 1970; Nguyen & Sampson, 1985).

As 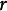 and 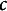 take on different possible values between 0 and 1, the set 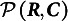 varies accordingly along points such as 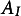 , 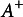 , and 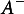 . This allows us to move from simply sampling from one line segment, produced by a certain point 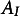 or Fréchet bounds 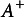 and 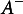 , to finding those for any given set of 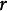 and 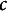 , and thereby obtaining various sets of 2 × 2 PMF matrices that conform to certain models or set constraints. By doing so, we can explore all parts of the tetrahedron that define the complete data space. In short, simply varying 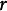 and 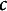 , without additional constraints, allows us to pick data points from any part of the space 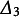 .

Constraints can also be added. For example, to evaluate the independence model, one could consider all points 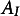 and thereby generate the hyperbolic paraboloid that forms the *surface of independence* shown in Figure 4 (Fienberg & Gilbert, 1970). For 2 × 2 tables, the surface of independence divides the simplex into two subsets: positively quadrant dependent (PQD) and negatively quadrant dependent (NQD) matrices (Nguyen & Sampson, 1985). Elaborating, 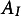 divides the line segment from 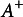 to 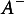 into two parts with segment 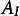 to 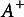 referring to the PDQ matrices and 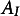 to 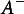 representing the NQD matrices for a certain 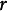 and 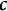 . When considering the entire tetrahedron in Figure 4, the PQD subset is the part of the simplex containing faces 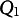 and 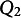 , and the NQD subset is the part containing faces 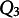 and 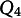 . The term PQD implies a positive association between 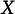 and 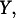 or items 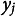 and 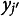 , while NQD indicates a negative association (Douglas et al., 1990). Defining association by the odds ratio, where 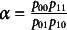 , 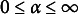 (Fienberg & Gilbert, 1970), the surface of independence exists for 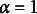 . If 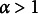 , the subset is strictly PQD, and if 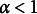 , strictly NQD. Note that this clean split of the data space into PQD and NQD subsets only applies to 2 × 2 tables, though the concept of quadrant dependence also applies to ordinal contingency tables with more than two categories (Bartolucci et al., 2001; Rao et al., 1987).

### 3.3 Geometry of m × n contingency tables with fixed margins

Generalizing to *m* × *n* contingency tables with fixed row and column marginal probability vectors of 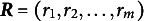 and 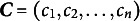 , the set 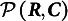 of all *m* × *n* PMF matrices 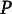 now consists of cell probabilities for an item pair that reside in the 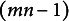 -dimensional simplex 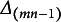 . In our context, 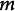 and 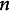 are the numbers of response categories of items 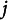 and 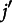 , respectively, and the dimension is 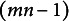 because the probability simplex is constrained by 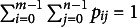 , so that one degree of freedom is lost. Every matrix *P* can thereby be represented by a point 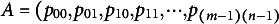 ) in 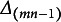 .

The set 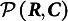 can be found as a subset of 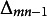 that satisfies a set of conditions laid out by the Fréchet bounds for any individual cell probability 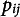 where 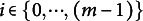 and 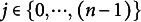 for all possible values of 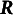 and 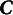 . The bounds for each cell independently are
(7)

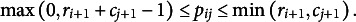



This results in hyperplanes that are bounded by the extreme matrices 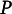 created by the Fréchet bounds and thus define the subspace of 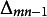 where valid data points may be found. If other constraints are added, then valid points will reside in even more constrained subspaces of 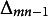 . As one example, if we consider only the points pertaining to the independence model, then 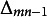 will be constrained to the *manifold of independence*, which is a generalization of the surface of independence to 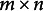 tables.

The geometric representation of contingency tables with fixed margins lays the theoretical foundation for a LI-based data-generating mechanism for one item pair. However, we still need to be able to randomly sample many contingency tables, corresponding to all unique item pairs within a set of items, simultaneously, and while conforming to specific marginal constraints. Although various methods are possible, we selected a sequential importance sampling (SIS) approach, which (1) offers efficiency in sampling multi-way tables of many rows and columns with fixed margins (Chen, Diaconis, et al., 2005), and (2) enables us to independently and randomly sample the contingency tables for each item pair.

### 3.4 Sequential importance sampling of contingency tables with fixed margins

SIS randomly samples probabilities from a target contingency table in a sequential manner. Each cell probability is a random variable, so the resulting contingency table is also a random variable. Suppose 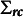 is the set of all *m* × *n* contingency tables with row marginal probability vector 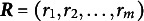 and column marginal probability vector 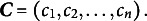 Let 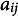 be the element at the 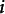 th row and 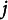 th column of a contingency table. The process of SIS begins with sampling one cell (e.g., 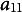 ) and filling in the remaining cells one-by-one, generally from column to column, to adhere to the probability constraints of contingency tables.

Recall that the necessary and sufficient condition for the existence of a contingency table of probabilities with 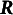 and 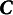 is
(8)

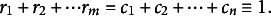



The sampling process begins with the first cell, 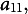 which needs to satisfy conditions
(9)

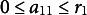

 and
(10)

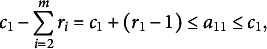

 which can be combined as
(11)

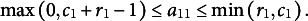



Note that this matches the Fréchet bounds for any cell probability 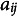 defined in Equations (4) and (5) (Chen, Dinwoodie, et al., 2005; Fienberg, 1999). Specifically, the Fréchet bounds determine the lower and upper limits of a bivariate probability based on the surrounding univariate margins, and 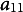 is randomly sampled from the uniform distribution between the lower and upper Fréchet bounds. We note that other distributions, such as the hypergeometric distribution (Johnson et al., 2005) and the conditional Poisson distribution (Chen, Diaconis, et al., 2005), can also be used for sampling, depending on the structure of the contingency table and corresponding assumptions.

The entire sampled contingency table is the result of sequentially fixing the free cell probabilities in the table (Fienberg, 1999; Nguyen, 1985) and calculating the remaining cell probabilities via marginal constraints. After sampling (and thus fixing) 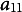 , the same logic is used to recursively sample the remaining free cells in column 1 ( 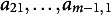 ) with each cell’s Fréchet bounds repeatedly updated to incorporate information from the previously sampled cell probabilities:
(12)

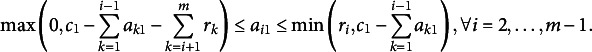



The final cell in column 1 ( 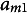 ) is straightforward to compute as it must satisfy the condition that the sum of cells in column 1 equals the column marginal 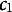 , such that 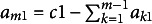 .

The same process then extends recursively, sampling the free cells in the subsequent columns ( 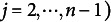 under constraints of their respective bounds, which are defined as
(13)

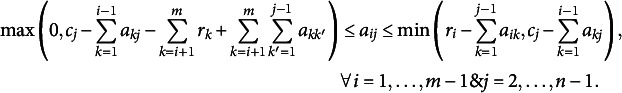

 The final cell in each column, 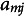 , is computed directly as 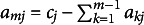 . Lastly, all values in the last column, ( 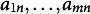 ), are fully determined by previously sampled values to ensure all marginal constraints are satisfied and calculated as 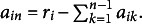 For the last cell, 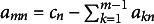 is equivalent to 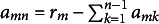 . We note that although we presented the logic by breaking down the process—initializing 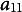 , iterating through remaining cells in the first column, and then moving on to subsequent columns for clarity—the same procedure applies across all columns. Equation (13) serves as the general form, naturally simplifying to Equation (12) for intermediate cells in column 1 and further reducing to Equation (11) for 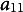 .

The process above highlights the distinction between free and pre-determined cells. In a two-way contingency table with marginal constraints, the number of free cells to sample is 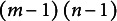 , which corresponds to the degrees of freedom. The remaining cells are not free but are straightforwardly calculated based on existing marginal information. For example, in a 2 × 2 table with given row and column sums (i.e., 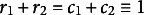 ), the degrees of freedom is 1, so a single cell probability (e.g., 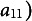 is the only variable that needs to be sampled from a uniform or hypergeometric distribution within the range of [ 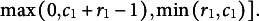 All other cells can then be filled as 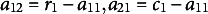 , and 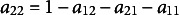 .

## 4 Sequential importance sampling algorithm to quickly and uniformly obtain contingency tables (SISQUOC)

### 4.1 Defining the complete data space

When considering only the first- and second-order marginal moments, the complete data space of item response patterns contains all possible bivariate margins that simultaneously satisfy the bounds set by all univariate margins (i.e., Fréchet bounds) across a set of items. Understanding the relationship between the simplex, the complete data space of two-way tables, and the Dirichlet distribution is foundational for uniformly sampling all valid two-way tables. By leveraging the geometry of the simplex and the flexibility of the Dirichlet distribution, it is possible to explore the entire data space of two-way tables under fixed or varying marginal constraints. For this, assume *J* items, where each item 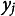 has 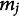 categories. Each unique item pair 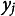 and 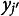 forms a 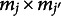 contingency table, where the cell probabilities are defined as 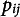 , where 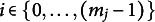 and 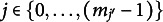 . 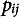 is the bivariate probability for ( 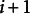 )th row and the ( 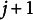 )th column. Each pairwise table must satisfy a set of constraints. All cell probabilities must be non-negative, meaning 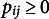 for all 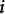 and 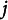 . Additionally, the row and column sums (marginals) are fixed and must follow 
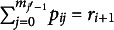
 and 
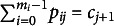
 , where 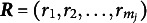 and 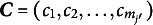 represent the row and column marginal probabilities, respectively. Finally, the total sum of all probabilities in the table must equal 1, such that 
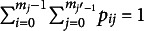
 .

Geometrically, for a set of fixed margins and Fréchet bounds, the data space forms a polytope within a 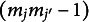 -dimensional simplex. By varying the margins, the complete data space becomes the union of all such polytopes, effectively spanning the overall simplex defined by all possible configurations of row and column marginal constraints. The Dirichlet distribution provides the mathematical framework for modeling and sampling from this data space. Widely used in IRT due to its connection to multinomial data, this distribution also underpins the geometry of 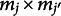 contingency tables as its probability density function (PDF) corresponds to the 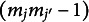 -dimensional simplex. The joint distribution of cell probabilities is given by 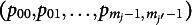 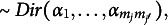 where the concentration parameters 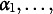 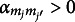 govern the shape of the distribution. Setting all 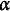 -parameters to 1 ensures uniform and random sampling across the data space, respecting the geometry of the simplex and imposed marginal constraints. Thus, for a 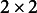 table, the data space corresponds to 
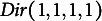
 uniformly covering all possible configurations.

### 4.2 Proposed data generation algorithm

To randomly and uniformly sample data points from the target space defined above, we follow a hierarchical approach consisting of three steps: (1) define a distribution for univariate margins and randomly draw univariate probabilities, (2) randomly sample bivariate probabilities arising from an item pair under the pre-generated univariate margin constraints for each item, and (3) do steps (1) and (2) while considering the lower-order margins of all possible unique item pairs at once. The contingency tables for all item pairs are not entirely independent as they can share some univariate margins with other contingency tables, depending on the item pair in question.

Starting with Step (1), the aggregation property of the Dirichlet distribution provides a robust and theoretically justified foundation for defining univariate margins, particularly for general two-way tables where all items share the same number of categories. When 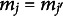 for all items, the large majority in research and the focus of this article, the univariate margins for the row and column variables are obtained by summing the concentration parameters across 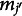 columns or 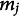 rows, respectively. With ( 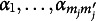 ) all set equal to 1, the distributions simplify to 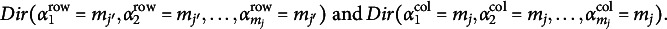 Consider once more a 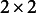 table following 
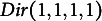
 . The univariate marginal distributions for the row and column variables (i.e., paired items) then become 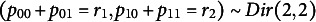 and 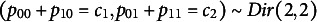 , both of which reduces to 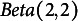 for two categories. The aggregation property ensures that the univariate marginal distributions remain consistent with the joint Dirichlet distribution, preserving the simplex geometry and uniformity of the data space for two-way tables with imposed marginal constraints. Research on the Dirichlet distribution, contingency tables, and simplex sampling (e.g., Diaconis & Efron, 1987; Letac & Scarsini, 1998; Lin, 2016) details their properties and supports their applications in the current modeling and sampling framework.

In less common cases where items have an unequal number of categories, univariate margins must satisfy differing constraints imposed by multiple pairwise tables. For instance, the univariate margin for a binary item appearing in both 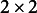 and 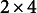 tables is influenced by 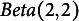 and 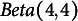 , respectively. These dependencies emerge naturally from the joint structure, meaning that the complete data space for a two-way table cannot be defined by a single marginal constraint. Mixtures of Dirichlet distributions provide a principled way to incorporate multiple constraints, blending each constraint in a weighted fashion to allow uniform and random sampling within the possible data space. Univariate probabilities are obtained from a mixture of Dirichlet distributions, with draws from each Dirichlet proportional to the relative contribution (i.e., weights) of item 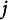 ’s univariate margin constraints across its ( 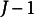 ) pairwise tables, as determined by the aggregation property. For instance, for the binary item above and assuming three total items, 50% of all univariate probabilities are drawn from 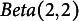 and 50% from 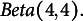 This process allows each constraint to shape the bivariate space relative to its contribution, ensuring that every valid contingency table of the defined data space is sampled with equal probability. Albert and Gupta (1982) and Good (1976) laid the theoretical foundation for using Dirichlet mixtures by highlighting their flexibility in modeling heterogeneous constraints in contingency tables. Aitchison (1985) also emphasizes the utility of mixtures in capturing complex relationships on the simplex.

**Figure 5:**
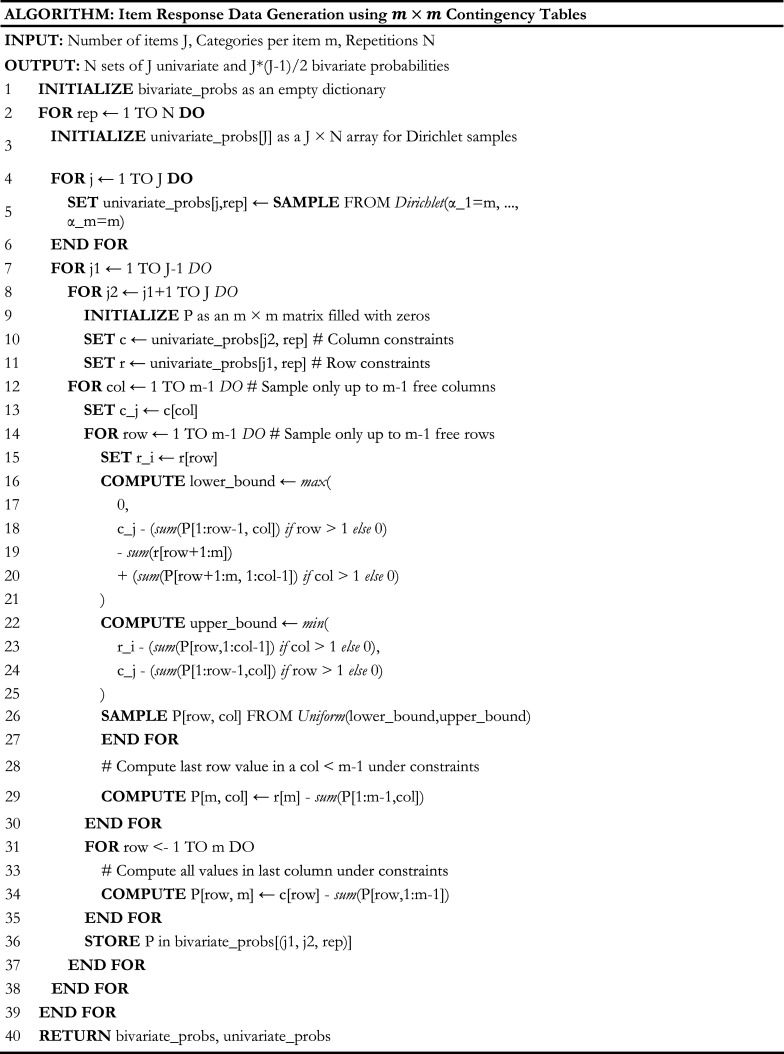
Proposed generalized data generation algorithm: SISQUOC.

**Figure A1:**
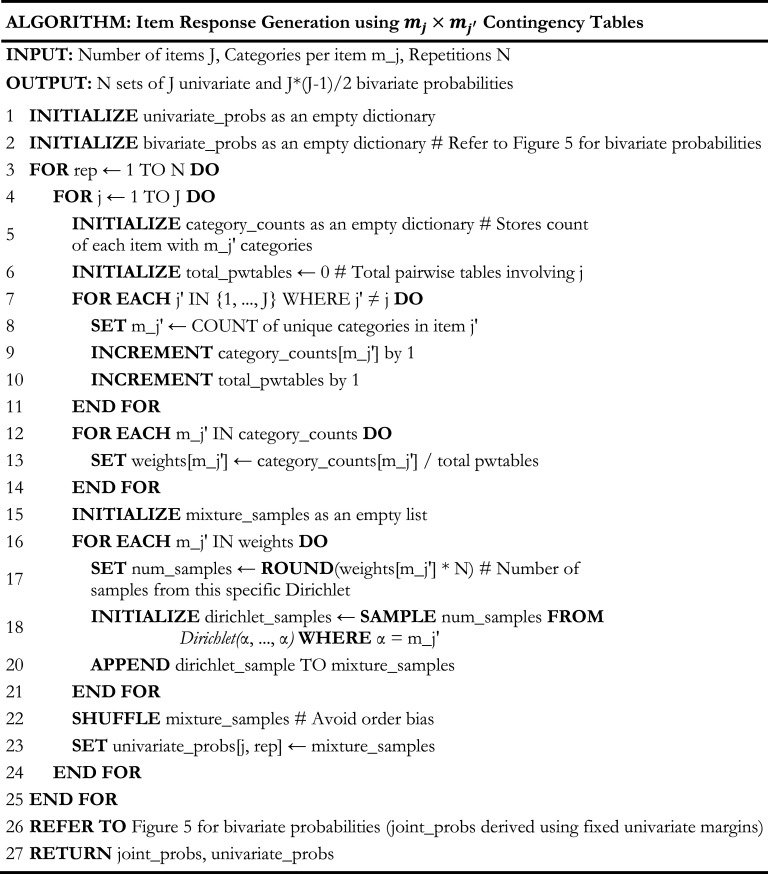
*Proposed data generation algorithm for* 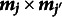 *tables (mixed-category items).* *Note:* The weight assigned to each Dirichlet component for item 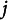 is given by 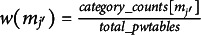 where 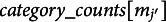 is the number of pairwise tables involving 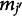 and 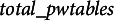 is the total number of pairwise tables for item.

Step (2) can be achieved by combining knowledge of the Fréchet bounds, which dictate the lower and upper bounds of a bivariate probability based on the surrounding univariate margins sampled from the Dirichlet distribution, and adapting the SIS method proposed by Chen, Diaconis, et al. (2005). For Step (3), our hierarchical approach first samples the respective univariate margins of each specific item pair based on the aggregation property to align with the imposed marginal constraints. Using SIS with fixed margins and Fréchet bounds, our method facilitates the independent sampling of bivariate probabilities for each contingency table, rather than requiring simultaneous sampling of all two-way tables. This enables us to address one item pair or contingency table, repeating the process for all unique item pairs while maintaining consistency across shared margins. Weaving these pieces together, we propose the data generation algorithm termed Sequential Importance Sampling algorithm to Quickly and Uniformly Obtain Contingency tables (SISQUOC). The process is outlined in Figure 5 for items with equal categories, based on the general Equation (13). An extension to mixed-category items, focused on univariate margins, is given in Figure A1.

SISQUOC can readily generate large quantities of dichotomous and polytomous item response data. For example, generating 50 items that each include four response categories requires a total of 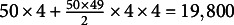 data elements. This number is within an easily manageable range for most computers. The same cannot be said if attempting to use the simplex sampling method, which requires generating 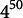 item response probabilities, which is greater than 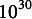 . The R code for our algorithm, along with examples, is available at https://github.com/ysuh09/SISQUOC.

### 4.3 Algorithm validation and performance assessment

**Figure A2:**
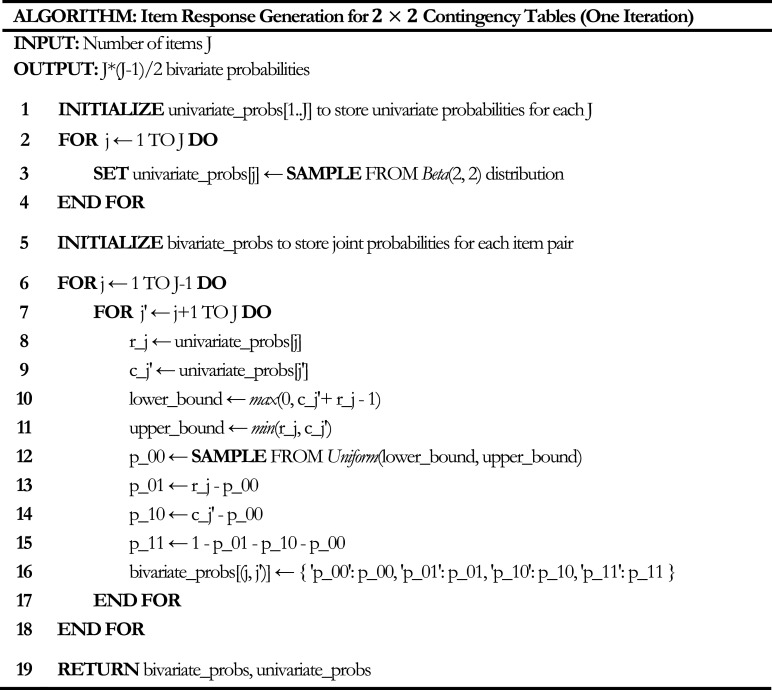
Proposed data generation algorithm for 2 × 2 tables.

Theoretically, our proposed algorithm should be able to sample uniformly and randomly from the desired categorical data space. To evaluate its performance, we compared our method to the simplex sampling method in Bonifay and Cai (2017) and the theoretical Dirichlet distribution, examining them graphically, statistically, as well as by studying their computational complexity^1^. For the graphical and statistical comparisons, we focused primarily on a single dichotomously scored item pair, thereby ensuring feasible visualization and a valid comparison between methods (see Suh (2022) for more detail). This was equivalent to sampling a 2 × 2 table, as depicted in Figure A2 in the Appendix, for one iteration, with the theoretical distribution 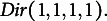 Regarding computational complexity and efficiency, we provide more generalized results that are applicable to the case of many items and/or multiple categories.

**Figure 6:**
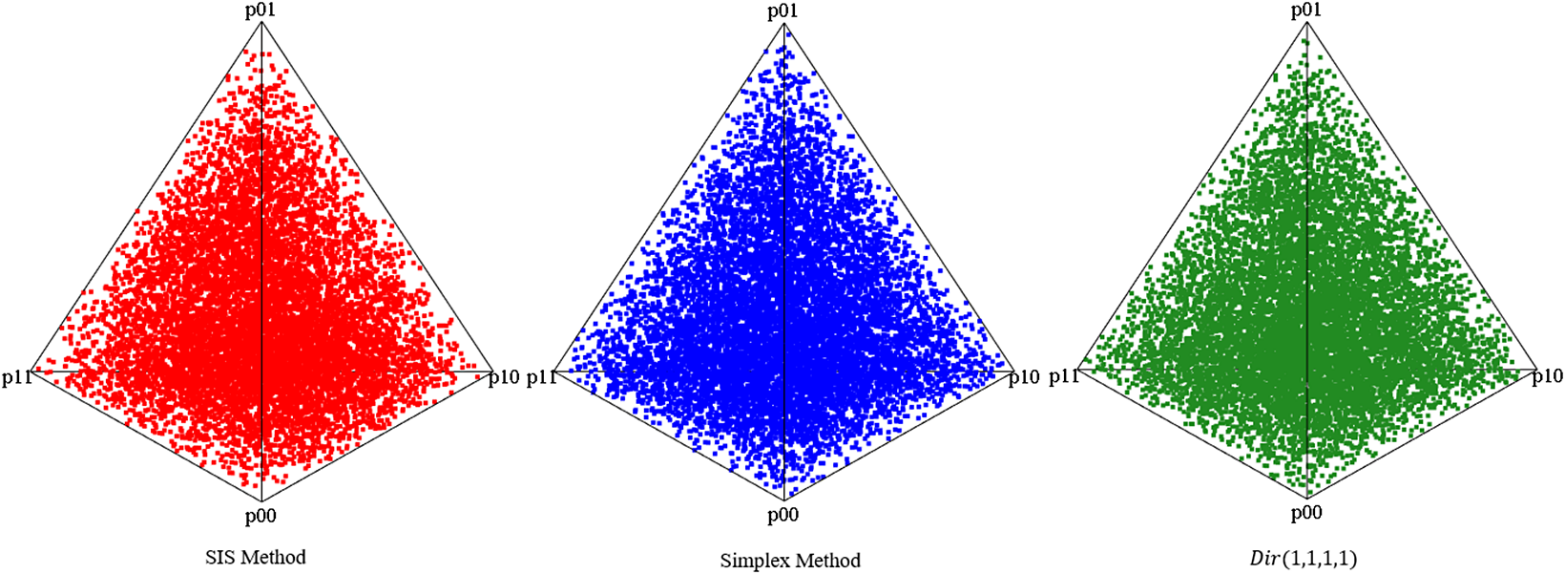
*Bivariate margins for SISQUOC, simplex sampling method, and* 
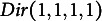
 .

**Figure 7:**
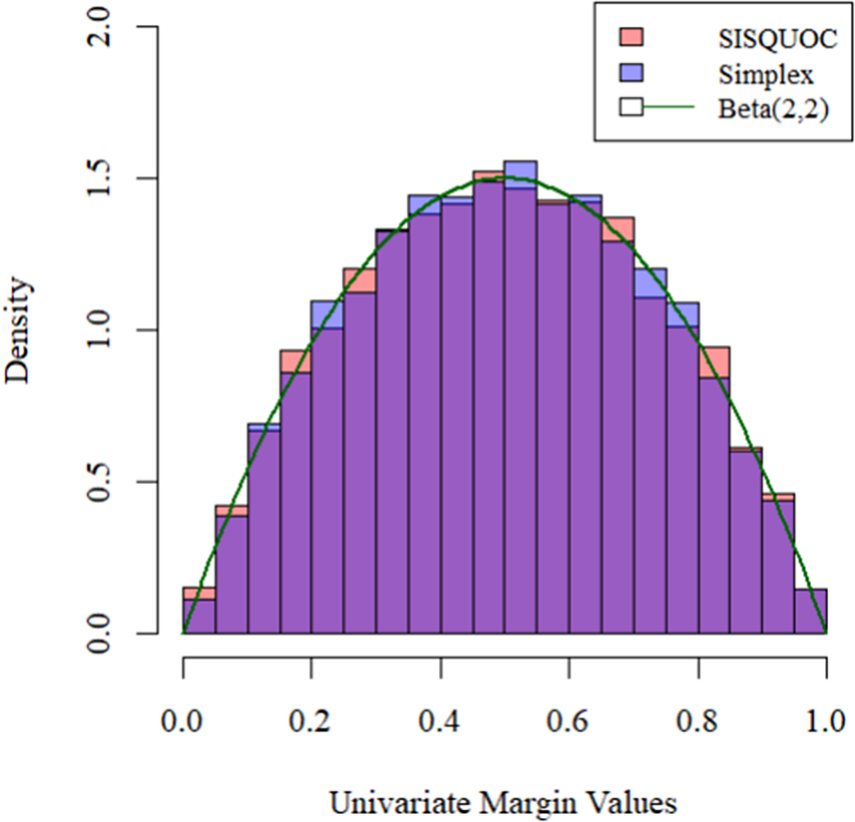
*Univariate margins for SISQUOC, simplex sampling method, and* 
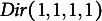
 .

In total, we sampled 10,000 contingency tables (bivariate points) using the proposed SISQUOC, simplex sampling method, and theoretical distribution. In Figure 6, 3D scatterplots (in which each point is a sampled 2 × 2 contingency table) provide a visualization of random uniform sampling of the entire data space. Graphical comparisons across the three methods show a clear alignment in the overall distributions of points. In Figure 7, histograms with 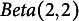 overlays for the univariate marginals further underscore the distributional similarity across methods. These visual findings are supported by descriptive statistics, which exhibited consistent means and variances across all methods (Table A2 in the Appendix). Figure 7, being a 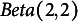 distribution, also demonstrates that uniform random sampling from the complete data space defined by bivariate and univariate margins is not equivalent to sampling individual items from a uniform distribution. This is further supported when plotting the bivariate margins, as simply multiplying items sampled from a uniform distribution would result in the distribution seen in Figure 4 rather than Figure 6.

We conducted several statistical tests comparing SISQUOC to the theoretical 
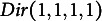
 distribution, focusing on uniformity and randomness in the complete data space (Tables A2 and A3 in the Appendix). The Kolmogorov–Smirnov (K-S) tests for each univariate margin’s distribution returned *p*-values higher than 0.05, indicating no significant differences in the univariate dimensions. The chi-squared test, conducted using averaged values over iterations from the target and theoretical samples, yielded a *p*-value of 0.20, further supporting the uniformity of the distributions (e.g., Li (2015)). Additionally, the Kullback–Leibler divergence was small (KL = 0.0002), demonstrating strong alignment in terms of distributional fit. The maximum likelihood estimates of the alpha parameters for SISQUOC were also close to 1, consistent with the theoretical distribution, further validating our method. We obtained similar statistical results when comparing the simplex sampling method to the theoretical Dirichlet distribution. Collectively, these results suggest that our method performs statistically comparably to both the simplex sampling method and the theoretical Dirichlet distribution.

Assessing computational complexity—specifically, the time and space requirements using Big-O notation (Arora & Barak, 2009)—of our proposed algorithm and the simplex sampling method by Bonifay and Cai (2017) reveals significant differences in computational efficiency and scalability. Let 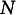 denote the number of iterations, 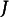 represent the number of items, and 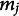 be the number of categories per item *j*. Our method demonstrates 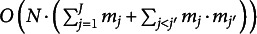 time and space complexity, while the simplex method operates with 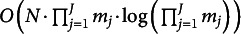 time complexity and 
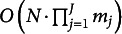
 space complexity. This comparison highlights the computational advantages of our approach in terms of both time and memory requirements. The quadratic complexity of SISQUOC ensures that the algorithm remains computationally feasible even as the number of items or categories increases, whereas the exponential complexity of the simplex sampling method restricts its scalability. Our approach is particularly advantageous in scenarios that demand efficient handling of high-dimensional data with significantly reduced computational burden.

Performance evaluations (Table A4 in the Appendix) corroborated these theoretical findings, where we tested both methods by varying the number of items 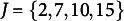 on a system equipped with an Intel Core i7-11800H CPU (16 cores, 2.30 GHz) and 32 GB RAM. While the experiments primarily focused on dichotomously scored items, their results generalize to the case of polytomous items as well. Across all tests, SISQUOC consistently outperformed the simplex sampling method in execution time, iterations per second, and memory usage. For even the smallest dataset of two items, the proposed method demonstrated a significant improvement, achieving up to 46 times faster execution and over 500 times more efficient memory usage compared to the simplex sampling method. In addition, as the number of items increased, the simplex sampling method exhibited exponential growth in both time and memory consumption, while our method maintained its quadratic scaling. These results are consistent with the theoretical complexity analysis and further confirm the superior efficiency of our proposed approach, particularly for larger datasets.

### 4.4 Lower-order margins and the iterative proportional fitting procedure

SISQUOC was motivated by the need to reduce the computational burden of generating item response data using a FI-based multinomial approach. The datasets simulated by our method only include information about the univariate and bivariate margins that can be used for estimation and subsequent model fitting. In IRT estimation, two primary approaches exist: the Underlying Variable (UV) approach, which assumes normally distributed latent traits and utilizes LI methods (e.g., polychoric correlation matrices), and the IRT Approach, which employs FI methods to directly model response probabilities (Cai & Moustaki, 2018). The UV approach can be used with our data generation method, but it is constrained in its ability to estimate complex IRT models compared to FI methods.

As discussed earlier, conventional FI-based estimation methods for IRT models require the full multinomial contingency table of item response patterns. Using the IPFP, we can reconstruct a joint distribution for such multinomial data that satisfies, as much as possible, the constraints of the bivariate marginal probabilities produced by SISQUOC. The IPFP was first proposed by Deming and Stephan (1940) to estimate cell probabilities in a contingency table, subject to marginal constraints. Since its conception, the IPFP has been applied to a variety of statistical problems by an equally diverse number of sources (Fienberg, 1970). Among other applications, it has been repeatedly used in simulating multivariate binary data subject to constraints of mainly fixed marginal distributions with specified degrees of association (e.g., Barthélemy & Suesse, 2018; Gange, 1995).

Let us assume *J* binary variables 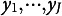 with success probabilities 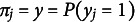 for 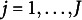 . As *J* grows larger, it becomes increasingly difficult to specify and determine 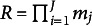 probabilities. An alternative is to specify the *J* probabilities 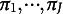 and 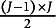 pairwise-probabilities 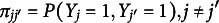 , and use the IPFP to find a solution of 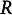 probabilities, where the marginal one- and two-dimensional probabilities satisfy { 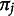 } and { 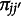 }. There are often many higher-order tables that have the same univariate and bivariate margins, so many solution datasets are possible. The IPFP ideally converges to one of these equally valid solutions. In comparison to other approaches toward the same goal, the IPFP has the advantage that it produces strictly positive joint probabilities, meaning that, theoretically, none of the 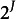 sequences can be excluded. Furthermore, it can simulate MVB distributions without assuming an underlying continuous (normal) model, so that imposed restrictions, such as positive definite correlation matrices, need not be met. This makes the IPFP especially attractive in the present context, as our aim is to randomly sample from the complete data space. Furthermore, our method is well-suited for IPFP because it generates pairwise probabilities directly, eliminating the need to transform traditionally used correlations or odds ratios to satisfy the marginal constraints required in the IPFP.

Using the IPFP in conjunction with our data generation method means setting the univariate and bivariate margins of simulated datasets as marginal constraints for the joint distribution of 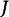 variables. We start from an array of size 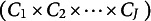 whose cells are all equal to 1. This is the simplest and most uninformative case, and starting from a different array would mean adding information that is not available (Ranalli & Rocci, 2016). Then, multiplying by appropriate factors, we adjust the cell probabilities of the joint distribution successively to match the probabilities for each bivariate table. The process is continued until convergence, defined as the difference in fitted probabilities between two consecutive iterations being less than an arbitrary 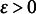 .

## 5 Example application: fitting propensity of polytomous item response models

Suh (2022) tested the suitability of the LI-based data generation method—coupled with model estimation using conventional methods under the multinomial framework, made possible by the IPFP—for investigating FP. Her findings supported the proposed methodology as a promising alternative to the method employed by Bonifay and Cai (2017). The computational feasibility gained from utilizing a LI method opens the door for examining the FP of many more models than before. As an example, we demonstrate the use of LI methods for FP evaluation of two polytomous IRT models.

### 5.1 Graded response model and generalized partial credit model

The choice between the graded response model (GRM; Samejima, 1969) and the generalized partial credit model (GPCM; Muraki, 1992) has been an ongoing topic of debate (as summarized below), driven by the fact that both models are equal in terms of the number of item parameters, but different in how their functional forms parameterize the category response probabilities. That is, the GRM and GPCM have the same parametric complexity, but may differ in configural complexity.

Let *J* items be measured for *N* individuals with 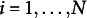 and 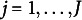 . Suppose 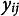 is the response from person 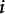 to item 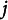 that has 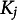 ordered categories consisting of 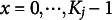 scores and assume unidimensionality for latent ability 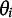 . In the GRM, the item response function is specified as a series of two-parameter logistic (2PL) item response functions:
(14)

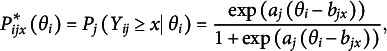

where 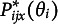 is the probability of person *i* with ability 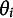 scoring 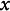 or above on item *j*, which is characterized by one slope parameter 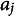 and a set of threshold parameters 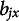 . By definition, 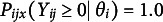 and 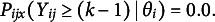 The probability of endorsing each of the remaining response categories is given by 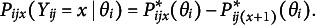


In contrast, the GPCM uses partial credit scoring, so the goal is to obtain the relative difficulty of each “step” required to transition from one response category to the next. The GPCM makes use of local or adjacent category logits and models the probability of obtaining a score of 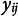 vs. 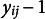 :
(15)

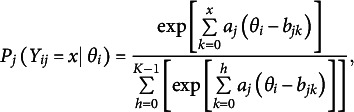

 where 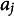 is the same as in the GRM and 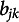 is known as the *k*th step parameter, representing the location along the latent trait continuum beyond which the respondent is more likely to receive a score of 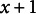 than a score of 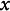 . Like the GRM, the GPCM involves 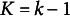 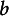 parameters per item *j.* Thus, the GRM and GPCM contain the same number of parameters, but their parameters are not directly comparable because their distinct functional forms reflect different ways of characterizing the response categories (Ostini & Nering, 2006).

While there is a clear understanding of the theoretical or mathematical distinctions between polytomous IRT models, the practical implications of selecting a GRM or GPCM remain relatively obscured (Bolt, 2002). Existing studies comparing the model fit of the GRM and GPCM are divided. Kang et al. (2009) generated data from different polytomous IRT models and explored a set of relative global fit indices. They found that when data were generated from the GRM, some indices supported the GPCM as a better fit than the GRM, but the reverse did not occur when data were generated from the GPCM. Such results suggest that the GPCM is more configurally complex than the GRM. Conversely, many application-based studies provide empirical evidence favoring the GRM over the GPCM (e.g., Maydeu-Olivares, 2005; Sischka et al., 2020). This may be because the data-generating process is indeed the GRM, or it could be that the GRM is more configurally complex than the GPCM. Other studies suggest that the differences between the two models are extremely hard to distinguish using GoF model comparison criteria (e.g., Bolt, 2002; Maydeu-Olivares et al., 1994). In fact, Maydeu-Olivares et al. (1994) showed that differences were minuscule in most cases and concluded that either model can be equally appropriate for most practical settings. This has led some authors to rely on anecdotal evidence; for example, Thissen and Wainer (2001) observed, “In our experience, fitting hundreds of datasets over two decades, it has almost always been the case that the graded model fits rating data better than does the generalized partial credit model” (p. 151). Here, we add to this debate by examining the FP of the GRM and GPCM, which could not be assessed previously because of the computational limitations of the simplex sampling method that prevented its generalization to study the FP of polytomous IRT models.

### 5.2 Fitting propensities: graded response model and generalized partial credit model

#### 5.2.1 Study design

Our design consisted of simulating item response data from seven items, each with four categories. Item response data were generated using the proposed SISQUOC, which was equivalent to sampling 4 × 4 tables for each of the 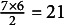 unique item pairs with fixed univariate probability constraints for each item. Following the algorithm in Figure 5, we first randomly sampled four univariate probabilities from a 
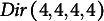
 distribution for all seven items. For each item pair, we started from the first cell of the first column and sampled the three bivariate probabilities in that column from a uniform distribution, updating the lower and upper Fréchet bounds as we sampled each cell. The last cell of the first column was directly calculated to satisfy the constraints of the univariate probability margin for that column. This process was repeated for the second and third columns. The bivariate cell probabilities for the fourth column were then directly calculated as their values were determined by cells that had already been filled. Each margin was multiplied by an arbitrarily chosen large sample size 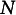 to obtain the number of sample responses rather than the response probabilities.

We simulated a total of 1000 random datasets, with each consisting of 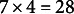 univariate margins and 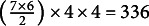 bivariate margins. The univariate and bivariate margins of the 1000 SIS-derived datasets comprised the marginal constraints for the joint distribution of 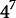 patterns using the IPFP. Thereby, all positive datasets contained the reconstructed full multinomial item response probabilities. We then used flexMIRT 3.65 (Cai, 2022) to fit the GRM and GPCM to each dataset via FI-based marginal likelihood estimation and the Expectation-Maximization (EM) algorithm. Following Bonifay and Cai (2017), we specified a more relaxed convergence tolerance of 0.001 for maximum parameter change in consecutive EM cycles and increased the maximum number of EM cycles to 20,000 to promote convergence.

To quantify test-level fit, we extended the Y2/*N* statistic (Bartholomew & Leung, 2002; Cai et al., 2006) to the polytomous IRT case:
(16)

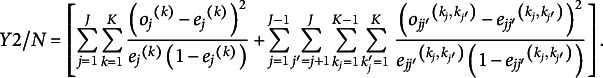



*N* is the sample size, *J* is the number of items, and *K* is the number of categories per item. 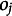 and 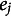 are the observed and expected linearly independent positive response frequencies for item *j*, and 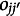 and 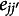 are the observed and expected linearly independent positive response frequencies for item pair 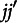 . We recorded the Y2/*N* indices from fitting both models to all datasets and analyzed them using empirical cumulative distribution frequency (CDF) plots and Euler diagrams (drawn using the *eulerr* package (Larsson, 2021)).

#### 5.2.2 Results

**Figure 8:**
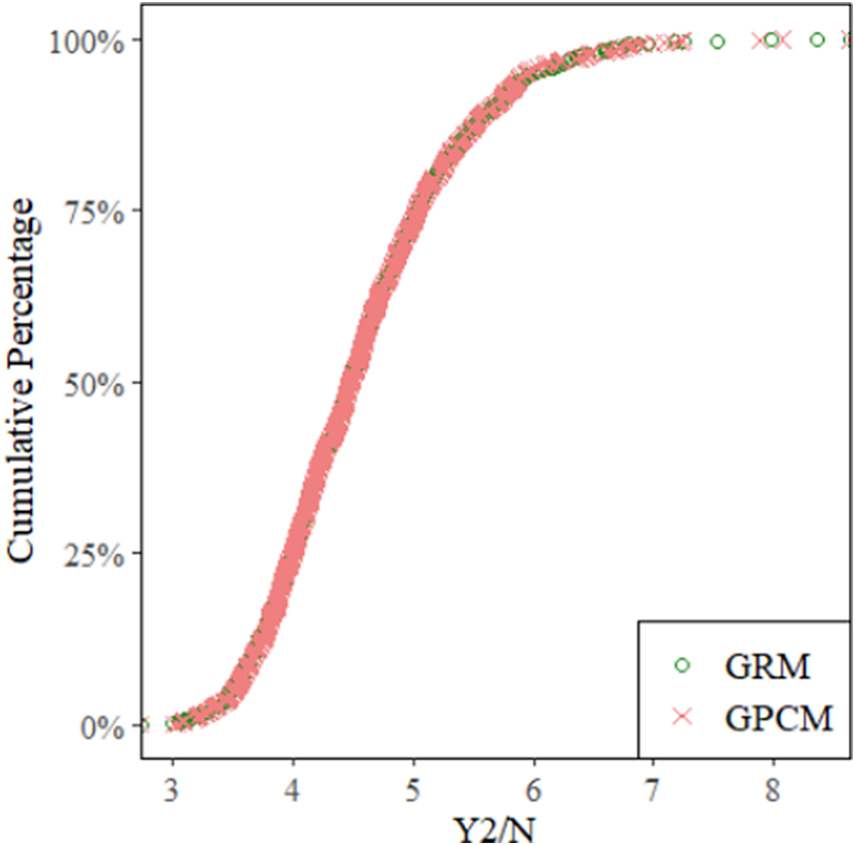
Cumulative percentage distributions of the Y2/N statistic.

Table 1 displays the Y2/*N* descriptive statistics for the GRM and GPCM across all 1,000 datasets. In terms of descriptives, the models were quite similar. This similarity is also evident in the empirical CDF plot (Figure 8), which shows the cumulative percentages of all datasets that achieved particular values of Y2/*N* for each model. The GRM and GPCM had nearly completely overlapping CDFs. Table A5 in the Appendix presents the deciles of the *Y2/N* values for each model, further establishing that these two models have nearly identical FP in terms of cumulative fit statistics.

**Table 1:** Descriptive statistics of Y2/N across all sampled contingency tables.

Model	Mean	SD	Min.	Max.
GRM	4.574	0.788	3.003	8.358
GPCM	4.578	0.78	3.012	8.072

*Note:* SD = standard deviation, Min. = minimum, Max. = maximum.

**Figure 9:**
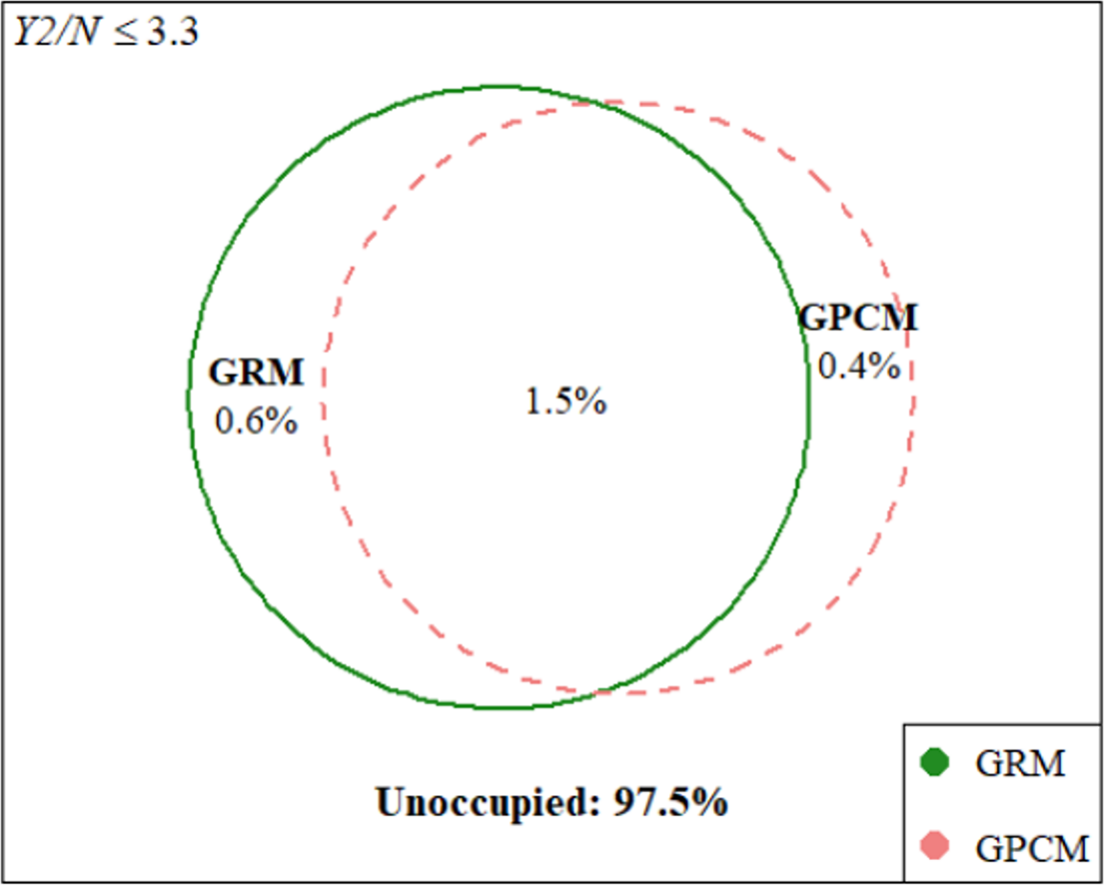
*Hypothetical approximate regions of the complete data space at* 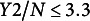 .

Importantly, empirical CDFs obscure an important aspect of FP: models with the same cumulative fit may correspond to non-overlapping regions of the complete data space. We can visualize these regions using Euler diagrams, wherein overlap between models indicates datasets for which both models satisfy a given Y2/*N* cut-point. We arbitrarily selected cut-points of 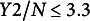 and 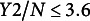 , as these values highlighted the shared and unique regions of the complete space that were occupied by the GRM and GPCM. As shown in Figure 9, with good fit defined as 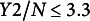 , the GPCM fit well to 19 of the 1000 datasets (i.e., 1.9% of the complete space). The GRM exhibited good fit to 15 of those 19 datasets, while also fitting well to 6 additional datasets, for a total of 21 datasets (i.e., 2.1% of the space).

**Figure 10:**
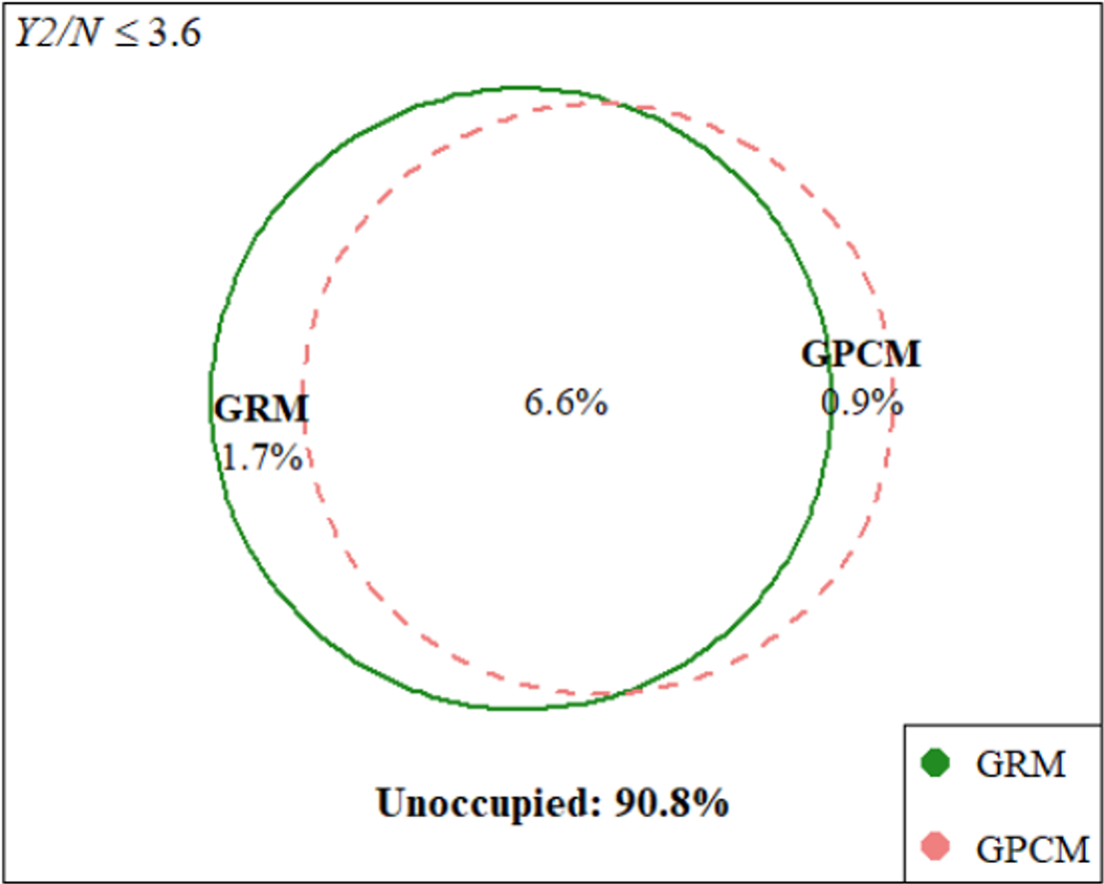
*Hypothetical approximate regions of the complete data space at* 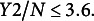

In Figure 10, where good fit is defined by 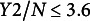 , we again see some divergence between the models: while both models fit well to 6.6% of the complete space, the GRM fit an additional and distinct 1.7% of the space, and the GPCM fit an additional and distinct 0.9%. This pattern of mostly shared and partially unique regions of the complete space persisted across increasing *Y2/N* cutoffs until the two models were completely overlapping.

Overall, these results indicate that the FPs of the GRM and GPCM are highly similar. The empirical CDFs of each model were essentially indistinguishable, implying that these models will fit well to approximately the same proportions of all possible categorical data. This finding supports previous claims (e.g., Maydeu-Olivares et al., 1994) that differences in fit between the GRM and GPCM are negligible. Yet, the Euler plots revealed small non-overlapping regions, indicating that each model can accommodate unique data patterns that the other cannot. Taken together, these findings imply that GoF statistics will not favor one of these models over the other, and that model selection should be aligned instead with the theoretical differences between the GRM and GPCM models (specifically, whether each response category is characterized relative to its adjacent categories or as part of a total score). Most importantly, this investigation into polytomous IRT model complexity was not readily possible with previously available methods.

## 6 Discussion and conclusion

Preacher (2006) introduced FP as a technique for quantifying model complexity (or parsimony) and demonstrated its usefulness in assessing configural complexity, which cannot be ascertained by counting the number of free parameters in a model. However, FP analysis requires data that represent the complete space of all possible data. In IRT modeling, progress in FP analysis has faced a roadblock: the simplex sampling method used in previous work (Bonifay & Cai, 2017) quickly becomes infeasible as the number of items and their response categories increase, but IRT analysis may involve many items and/or response categories within items, and thus requires a high-dimensional data space. Inspired by LI methods (Bolt, 2005), we proposed a novel algorithm, SISQUOC, that generates random datasets for IRT models using solely the univariate and bivariate moments. Our intent was to alleviate the computational burden associated with the exponential increase in the number of data patterns that are needed for FP analysis in the categorical data space. In turn, this will expand the utility of model complexity metrics as a means for model evaluation.

Our method combines classical work on the sampling of *m* × *n* contingency tables with fixed margins (e.g., Fienberg, 1999) and a SIS algorithm capable of sampling multi-way tables with many rows and columns (Chen, Diaconis, et al., 2005). The proposed SISQUOC was theoretically sound for both dichotomous and polytomous categorical data. We also compared our algorithm to the simplex sampling method used by Bonifay and Cai (2017) and demonstrated that both methods provided near-identical results and adequate coverage of the complete (tetrahedral) data space. The combination of LI methods and the SIS algorithm made simulating item responses computationally efficient and simple to implement. To that end, we verified that our method enables fast and easy generation of large quantities of random dichotomous and/or polytomous item data. In addition, this study also presented a method based on the IPFP that can recover the joint probabilities that satisfy the marginal probabilities generated through SISQUOC. Consequently, researchers can examine FP by applying traditional FI maximum likelihood methods alongside our proposed data generation method.

We also illustrated the use of SISQUOC by examining the FPs of the GRM and GPCM polytomous IRT models. For polytomously scored items, the data patterns to be generated can rapidly exceed manageable levels, thus making a LI approach particularly beneficial. The FPs of the GRM and GPCM were of special interest as these models have equal parametric complexity (i.e., an equal number of parameters) but may vary in configural complexity (i.e., due to their different functional forms). Results implied that these models have almost identical FPs in terms of cumulative fit statistics, though each model did occupy a small yet distinct area of the complete space that the other model did not. In general, these results align with past studies in which the two models produce very similar, if not indistinguishable results, especially regarding GoF model comparison criteria (e.g., Bolt, 2002; Maydeu-Olivares et al., 1994).

This work has various limitations and implications for future research. Regarding FP evaluation, the computational feasibility gained from using the proposed LI method instead of its FI counterpart opens the door for examining the FP of many more models than before. Such models may consist of many items and/or factors and multiple response categories per item. Considering the rise in large-scale IRT analysis, with tests that consist of many questions and involve complicated scoring methods, we expect that the computational tractability of LI methods will become increasingly favorable. Our example application with polytomous IRT models, by progressing FP analysis beyond the simple dichotomous case, is just one step in this direction.

The aim of this article is to unlock the potential that an LI approach may afford, and while SISQUOC is well-supported by both empirical results and theoretical justification, there is still room for further development. Moreover, it may not be the only method for uniform and random sampling from a complete data space defined by lower-order margins. Sampling from the unit simplex, a fundamental part of FP analysis for IRT, has been an active area of research due to its broad applications beyond IRT. Several methods have been proposed, including various exact sampling techniques and iterative approaches such as Markov Chain Monte Carlo (e.g., Diaconis & Sturmfels, 1998; Smith & Tromble, 2004). We selected the SIS method for several key reasons: SIS is well-suited for handling marginal constraints and efficiently navigating the geometry of discrete data spaces, such as those defined by lower-order moments and margins. This makes it ideal for contingency tables and LI models, where only partial data are available or lower-order marginals are the primary focus. Additionally, its simplicity and ease of implementation make SIS computationally lighter and more accessible compared to potential LI-based data-generating methods that use more complex approaches. Further exploration of SIS and other sampling methods could enhance overall sampling efficiency and illuminate specific use cases, especially in the case of mixed-category items. Comparative studies on the strengths and weaknesses of each method could help identify scenarios where one approach may outperform another, allowing researchers to build on the strengths of each. Additionally, future work could consider the inclusion of higher-order moments, which may capture more relevant characteristics of the data distribution and enhance its closeness to an FI-based approach. The costs and benefits of doing so, and whether it is ultimately worthwhile, remain to be determined.

It is also important to exercise caution when applying the presented LI-based method to FP analysis, particularly when interpreting FP results. For instance, in our comparison of polytomous IRT models, the superimposed CDFs and substantial overlap in the Euler plots do not imply that the choice between the GRM and GPCM is irrelevant. As shown in Bolt (2002), the GRM and GPCM can have similar GoF, but model misspecification can have severe implications for other aspects of model selection and use, such as DIF analysis. Samejima (1996) proposed multiple additional criteria for evaluating polytomous IRT models, stressing in particular that the model assumptions must match the cognitive situation, and that researchers must prioritize the theory and logic behind the model. We believe that the non-overlapping regions of the Euler plots in Figures 9 and 10 support such reasoning. In sum, FP results should be considered as part of a broader evaluation of model performance and suitability, rather than being the sole determinant in model selection.

Also, as IRT models grow increasingly complex and large, the need for estimation methods that efficiently leverage the univariate and bivariate margins from our data simulation approach increases. While the IPFP is highly capable, its extra step can be cumbersome, and computational limitations persist. LI-based estimation methods are well-established in the UV approach, such as pairwise likelihood estimation by Katsikatsou et al. (2012) for item factor analysis, which builds on composite likelihood methods (Varin et al., 2011). Similar methods, especially those developed based on the IRT approach, would offer greater flexibility and broader applicability across IRT models compared to the UV approach, while also integrating seamlessly with our data generation process. Initial results have been promising (Suh, 2022), both as a standalone estimation method and in applications to FPs with ongoing efforts to further refine and extend its utility in FP analysis and other IRT contexts.

Finally, our work paves the way to multiple new research areas. First, future work should study the flexibility and applicability of our data generation procedure (SISQUOC) to a wider range of models for categorical data. Although initially developed with IRT in mind, SISQUOC is a versatile, general-purpose algorithm that can be applied to a wide variety of contingency tables beyond just IRT models. For instance, rather than considering the complete data space, one could tailor our procedure to focus on different subregions thereof, generating data patterns ranging from theoretically possible to empirically plausible (Roberts & Pashler, 2000). Specific examples include the PQD subset and the surface of independence described earlier. In addition, the univariate and bivariate data generated through our procedure can be used to simulate other types of random data (e.g., polychoric correlation matrices, higher-dimensional multinomial data), which not only increases the range of latent variable models we can explore, but also presents the data in what may be a more familiar format.

Second, our LI-based approach also has implications beyond its use in FP investigation. For example, our suggested method can provide insights into the trade-off between statistical and computational efficiency. Although the loss of information on higher-order margins could theoretically hinder statistical efficiency, LI methods can be both appropriate and preferable when the computational efficiency gains significantly outweigh this loss. Moreover, the impact on efficiency may not always be substantial. For example, Katsikatsou et al. (2012) examined the bias and efficiency of pairwise estimation methods for item factor models and found these approaches to be adequate in practice. Building on such work, LI methods could be compared to FI methods to determine whether the information contained in the higher-order margins has a meaningful impact on the estimated parameters and/or model fit. This is especially relevant as our data generation method readily accommodates extensions to higher-order marginals. Combined with the fact that LI methods can be decomposed into simple additive components (Cai et al., 2006), this flexibility facilitates a systematic examination of the contribution of each margin to parameter identification and model misfit. This decomposition is particularly valuable in FP analysis: The data are generated with no *a priori* underlying structure, so issues of model misfit or misspecification are inevitable. Whereas studies suggest that the impact of model misspecification differs between lower- and higher-order margins (e.g., Hausman, 1978), and the ability to detect different types of misfit can vary depending on the margins examined (Li & Cai, 2018), research in this area remains sparse. This gap underscores the need for more comprehensive studies to fully understand the effects of margin-level misfit and improve the detection of model misspecification across different statistical methods, to which our work can contribute.

## Footnotes

1 In this section, we use the term “complexity” as it has been customarily used in theoretical computer science, e.g., in Big-O notation (Arora & Barak, 2009) and computational efficiency (via benchmarking).

## Appendix

**Table A1:** Cells and margins representations for a 2 × 2 contingency table.

Cells representation			Margins representation			
						
						
						
						

*Note:* Adapted from Maydeu-Olivares and Joe (2014).

**Table A2:** Maximum likelihood estimates and descriptive statistics of data generation methods.

Method	Dimension	Parameters	Mean	Variance	Skewness	Kurtosis
						
		Multivariate					
				0.251	0.036	0.769	2.877
				3.03	0.249	0.036	0.759	2.817
				3.06	0.250	0.037	0.775	2.870
			Marginal components	3.06	0.251	0.036	0.771	2.880
					0.5	0.051	0.007	2.131
SISQUOC	Univariate margins			2.05	0.501	0.049	– 0.004	2.156
						
		Multivariate					
					0.252	0.038	0.857	3.079
					3.03	0.249	0.038	0.870	3.075
					3.06	0.249	0.037	0.844	3.045
				Marginal components	3.06	0.250	0.037	0.860	3.123
					0.5	0.05	0.007	2.14
Simplex Sampling			Univariate margins		1.99	0.501	0.05	−0.027	2.123
						
		Multivariate					
			Marginal components		0.25	0.038	0.861	3.095
Theoretical		Univariate margins		0.5	0.05	0	2.143

*Note:* The theoretical distribution refers to a Dirichlet distribution,  . The marginal distribution is  where  . The univariate margins (summing over subsets such as  and  ) follow a  distribution. In our specific use case, the data should follow theoretical distributions of  ,  , and  , respectively.

**Table A3:** Statistical tests for algorithm validation for SISQUOC and simplex sampling method.

Method	Test	Statistic	*p*-Value
		0.014	0.258
		K-S test (univariate margins)	0.01	0.758
	Chi-squared test ( )	1.633	0.201
SISQUOC	KL divergence	0.0002	
			0.01	0.652
				K-S test (univariate margins)	0.019	0.058
			Chi-squared test ( )		1.426	0.232
Simplex sampling		KL divergence	0.0001	

*Note:* K-S test = Kolmogorov–Smirnov Test, df = degrees of freedom, KL divergence = Kullback–Leibler divergence. Both the SIS and simplex sampling methods were compared to the theoretical distributions [i.e.,  and

**Table A4:** Algorithm performance evaluation for SISQUOC and simplex sampling method.

	No. of	No. of	Min.	Median	Q1–Q3		Mem.	Total
Method	items	patterns	time	time	(IQR)	Itr./Sec	alloc.	time
					25.8–31.2			
		2	3	22.3	2.78	(5.4)	32,231	7.48 KB	31 ms
							107.1–233.7			
		7	28	92.1	116.1	(126.6)	6,186	71.07 KB	162 ms
					172.4–222.4			
	10	55	154.1	186.8	(49.98)	4,732	139.67 KB	211 ms
					359.5–418			
SIS-QUOC	15	120	331.2	378.1	(58.53)	2,493	304.94 KB	400 ms
					840.2–1031			
	2	4	756	903.6	(191.5)	998	3.82 MB	1 s
					982.4–1163			
		7	128	873.9	1110	(180.9)	907	3.84 MB	1.1 s
							1315–1404			
		10	1,024	1087	1340	(88.4)	728	3.99 MB	1.37 s
					8359–9284			
Simplex sampling	15	32,768	7000	8630	(925.6)	113	10.45 MB	8.83 s

*Note:* Min. = minimum, Q1 = 25% percentile, Q3 = 75% percentile, IQR = interquartile range. Itr. = iteration, Mem. alloc = memory allocation. Unless specified, default unit of time is   = microseconds =  seconds, ms = millisecond =  seconds, s = seconds. KB = kilobytes, MB = megabytes. Number of iterations = 1,000.

**Table A5:** Y2/N values at certain percentages of fitted datasets.

Deciles	0%	10%	20%	30%	40%	50%	60%	70%	80%	90%	100%
GRM	3.003	3.655	3.902	4.09	4.284	4.482	4.664	4.907	5.173	5.649	8.358
GPCM	3.012	3.676	3.906	4.097	4.27	4.481	4.659	4.907	5.203	5.652	8.072
